# Detection of Copper(II) Ions Using Glycine on Hydrazine-Adsorbed Gold Nanoparticles via Raman Spectroscopy

**DOI:** 10.3390/s16111785

**Published:** 2016-10-26

**Authors:** Nguyễn Hoàng Ly, Chulhun Seo, Sang-Woo Joo

**Affiliations:** 1 Department of Chemistry, Soongsil University, Seoul 156-743, Korea; nguyenhoangly2007@gmail.com; 2 Department of Information Communication, Materials, and Chemistry Convergence Technology, Soongsil University, Seoul 156-743, Korea; chulhun@ssu.ac.kr

**Keywords:** Cu(II), Raman spectroscopy, C≡N stretching mode, gold nanoparticles, glycine, intracellular imaging

## Abstract

A facile, selective, and sensitive detection method for the Cu^2+^ ions in environmental and biological solutions has been newly developed by observing the unique CN stretching peaks at ~2108 cm^−1^ upon the dissociative adsorption of glycine (GLY) in hydrazine buffer on gold nanoparticles (AuNPs). The relative abundance of Cu species on AuNPs was identified from X-ray photoelectron spectroscopy analysis. UV-Vis spectra also indicated that the Au particles aggregated to result in the color change owing to the destabilization induced by the GLY-Cu^2+^ complex. The CN stretching band at ~2108 cm^−1^ could be observed to indicate the formation of the CN species from GLY on the hydrazine-covered AuNP surfaces. The other ions of Fe^3+^, Fe^2+^, Hg^2+^, Mg^2+^, Mn^2+^, Ni^2+^, Zn^2+^, Cr^3+^, Co^2+^, Cd^2+^, Pb^2+^, Ca^2+^, NH_4_^+^, Na^+^, and K^+^ at high concentrations of 50 µM did not produce such spectral changes. The detection limit based on the CN band for the determination of the Cu^2+^ ion could be estimated to be as low as 500 nM in distilled water and 1 µM in river water, respectively. We attempted to apply our method to estimate intracellular ion detection in cancer cells for more practical purposes.

## 1. Introduction

Nanomaterial-based tools have been successfully implemented to monitor heavy metal ions [1]. By using nanomaterials, surface-enhanced Raman scattering (SERS) has provided one of the most sensitive methods on noble metal surfaces [2,3]. Selection of suitable reducing reagents is significant in the preparation of well-dispersed particles to study nanometric objects [4]. SERS has opened up novel opportunities in analytics to provide a vibrational fingerprint with high spectral resolution [5]. Nanoparticles have been used to detect a trace amount of analytes [6,7]. Various techniques have currently been developed for the imaging of the metal ions [8]. Inductively coupled plasma mass spectrometry is practically a powerful method that has been developed for metal ion detection [9]. However, it remains challenging to develop a novel analytical method for cheaper and easier metal ion monitoring.

At elevated concentrations, Cu^2+^ ions are toxic in fundamental biological processes and can cause damage to organs such as the liver and kidneys [10]. This potential hazard makes it indispensable to monitor the amount of copper in environmental water. Toward alleviating this issue, the selective detection of Cu^2+^ ions in aqueous solutions with high sensitivity is of great importance. To ensure safety, a detection limit as low as 20 µM (1.3 ppm) is recommended by the Environmental Protection Agency (EPA) [11]. Recently, ZnO@ZnS core-shell nanoparticles [12] and fluorescent gold nanoclusters [13] have been introduced to achieve efficient and selective detection of Cu^2+^ ions. Functionalized gold nanoparticles (AuNPs) [14] were also used for the detection of metal ions including Cu^2+^. SERS has also been used to discriminate Cu^2+^ ions in aqueous solutions with an interference of Hg^2+^ and Co^2+^ [15,16].

Glycine (GLY), one of the simplest amino acids, was known to adsorb on metal surfaces [17,18] via the carboxylate or the amino groups. GLY was predicted to bind the Cu^2+^ ion as a dimeric form [19]. Using an infrared spectroscopy tool, GLY was reported to dissociate to produce the cyanide group under alkaline pH conditions, when a positive voltage was applied to a gold electrode surface [20]. Cyanide bands at ca. 2100 cm^−1^ have been utilized as a marker band because of their discriminating position in the vibrational spectral window without the interference of multiple overlapping bands between 1000 and 1700 cm^−1^ [21,22]. In view of such electrochemical reactions [20], GLY may provide a unique marker of the cyanide group on metal surfaces.

In this study, we report a new Cu^2+^ ion detection method by means of SERS of GLY on AuNPs. UV-Vis absorption and a colorimetric method are also introduced to check the Cu^2+^-induced surface change. The variation of surface charge is characterized by means of zeta potential measurements. The cyanide band became prominent upon the increase of the Cu^2+^ ion in the GLY complex. This is the first study based on the selective detection of the Cu^2+^ ion using the dissociative adsorption of GLY on AuNPs that produces cyanide peaks whose intensities may be correlated with the Cu^2+^ ion concentration. Moreover, micromolar sensitivity could be achieved without the interferences of 15 ionic species including Hg^2+^ and Co^2+^ observed in the previous Raman studies [15,16]. These results may be useful in the development of a novel detection scheme to improve the efficacy of conventional methods. This study aims to develop a simple and inexpensive discrimination method for Cu^2+^ ions by applying AuNP-based sensors.

**Figure 1:**
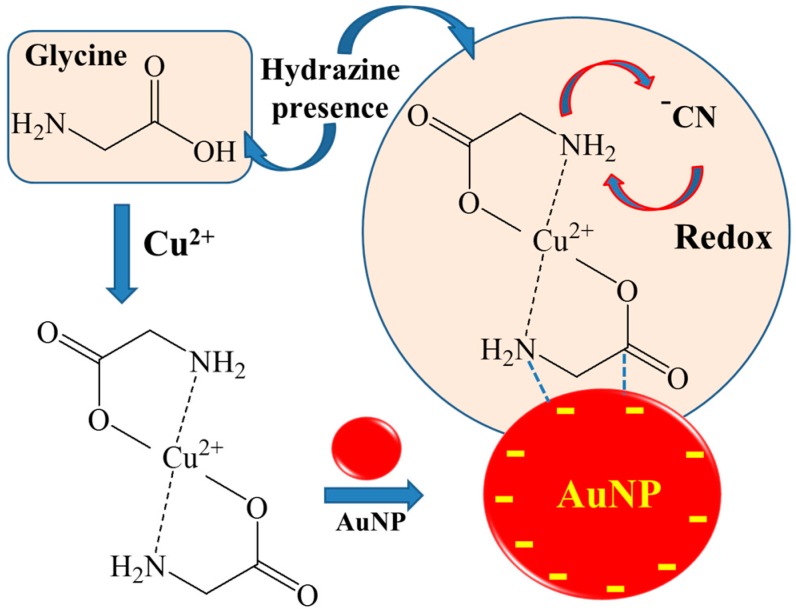
Schematic diagram of detection of Cu^2+^ ions due to the redox properties to lead the dissociation of GLY into the CN group on AuNP surfaces in the presence of hydrazine. The GLY-Cu^2+^ complex based on reference [19]. The increase of [Cu^2+^] results in more positive redox properties of the GLY-Cu^2+^ complex and stronger CN intensities.

Figure 1 shows our experimental scheme to detect Cu^2+^ ions with the complex of GLY in the presence of the hydrazine buffer, and subsequent adsorption on citrate-coated AuNPs. A dissociative reaction of GLY was found at higher concentrations of copper ions. By assembling and annealing the Cu^2+^ and GLY first in the hydrazine buffer, the complex can be ready to adsorb on AuNPs. We found that the Cu^2+^ ion made the surface charge of AuNPs more positive. On positively charged Au surfaces, GLY was dissociated to produce the cyanide ions, which were adsorbed on AuNPs. These reactions occurred under alkaline hydrazine buffer conditions at pH ~9.2. As control experiments, we tested the different amino acid such as alanine instead of GLY and performed independent trials using hydrazine, GLY, and NaOH with the same concentration and the pH value of ~9.2 without the commercial hydrazine buffer containing 0.6 M GLY, 0.5 M hydrazinium sulphate and 0.0375% chloroform. The CN peaks could not be observed without either GLY or the commercial hydrazine buffer. We found that the CN peaks decreased significantly after 99.999% nitrogen-gas bubbling for 10 min, although not shown here. Despite the possible existence of the oxygen species, the ambient air may affect the redox reactions to produce the CN peaks under our experiment conditions.

## 2. Experimental Section

### 2.1. Materials

GLY buffer solution (0.6 M, pH 9.2, Catalogue #G5418) containing 0.5 M NH_2_NH_2_ and 0.0375% chloroform and the other ionic (mostly nitrate) substances of Cu(NO_3_)_2_, Fe(NO_3_)_3_, Fe(C_2_O_4_), Hg(NO_3_)_2_, Mg(NO_3_)_2_, Mn(NO_3_)_2_, Ni(NO_3_)_2_, Zn(NO_3_)_2_, Cr(NO_3_)_3_, Co(NO_3_)_2_, Cd(NO_3_)_2_, Pb(NO_3_)_2_, Ca(NO_3_)_2_, NH_4_NO_3_, NaCl, and KNO_3_, as the forms of either the hydrates or the nonhydrates, were purchased from Sigma Aldrich (St. Louis, MO, USA). GLY (99%) and hydrazine solution (35 wt % in H_2_O) were also obtained from Sigma Aldrich. Human cervical carcinoma HeLa (ATCC^®^ CCL-2) cells were grown on Dulbecco Modified Eagle Medium in a steri-cycle CO_2_ incubator (Thermo Fisher Scientific) in 5% CO_2_/95% humidified air at 37 °C. Cell culture media were supplemented with 1% penicillin–streptomycin antibiotics (Gibco)/0.2 ppm plasmocin and 10% fetal bovine serum (FBS).

### 2.2. Instrumentations

We performed dark-field microscopy (DFM) [23] in cancer cells to check the possibility to apply our method under physiological conditions. We used the zeta potentials, transmission electron microscopy (TEM), energy dispersive X-ray analysis (EDAX), UV-Vis absorption spectroscopy, and SERS to characterize the AuNP suspensions. UV-Vis absorption spectra of the AuNP suspension were used to observe spectral changes with a Mecasys 3220 PC spectrophotometer. Surface charges were measured by zeta potential kits (Otsuka ELZ-2). High-resolution (HR)-TEM (JEOL JEM-3100) was used to observe the morphology of the AuNP Aggregates. EDAX spectrometer measurements were performed to find the Cu species assembled on Ni-coated grids by GLY-Cu^2+^ on metal nanoparticles using a Tecnai TF30 ST field emission TEM (300 kV). X-ray photoelectron spectroscopy (XPS) was conducted using a Sigma Probe Instrument Thermo VG spectrometer with an X-ray source of monochromic Al X-ray sources (Al Kα line: 1486.6 eV). The resolution of the full width at half-maximum is 0.76 eV in the Au 3d_5/2_ peaks.

### 2.3. Preparation of AuNPs and GLY-Cu^2+^ Complex

We found that the 40 nm AuNPs (Catalogue # EMGC40/4) from BBI solutions (Cardiff, UK) would show the strongest CN species under our experimental conditions. For the UV-Vis experiment, in the first step, GLY (0.6 M, 10 µL), Cu^2+^ (1 mM, 50 µL), and ultrapure water (800 µL) were put into a 1.5 mL Eppendorf tube, stirred and stabilized over 60 min at room temperature. In the second step, 140 µL of AuNPs was added to the GLY-Cu^2+^ complex. Excessive amounts of GLY and hydrazine in the sample solutions were not found to disturb the presented ion detection scheme.

For the zeta potential experiment, 10 µL Cu^2+^ (0.1 µM) and 50 µL GLY (0.6 M) were mixed and stirred for over 60 min at room temperature. Subsequently, 140 µL of the AuNP solution was added to the sample mixture and the surface charges of AuNPs appeared to shift toward the positive value after the addition of 10 nM of the Cu^2+^ ion by measuring zeta potentials.

1.0 mL of the resulting mixture solution was placed in a 1.5 mL centrifugal tube for the XPS experiment. The AuNPs-GLY-Cu^2+^ sample was collected by 4 °C centrifugation at 10,000 rpm for 10 min. After the supernatant parts were removed up to a residual volume of 20 µL, the 20 µL sample of AuNPs-GLY-Cu^2+^ was dropped on a 5 mm × 5 mm cover glass purchased from Deckgläser (Sondheim/Rhön, Germany). The sample was then dried at 75 °C overnight.

For the SERS experiment, 10 µL of Cu^2+^ (1 mM, in either distilled water or river water) and 50 µL of GLY (0.6 M, GLY buffer solution, pH 9.2) were mixed and stirred at room temperature for over 1 h. Subsequently, 140 µL of AuNPs was added to the mixture to examine the adsorption of GLY-Cu^2+^ on AuNPs by recording the SERS spectra of the samples. The pH value was measured to be 8.9 in all the SERS measurements. Since we had used the GLY buffer solution with the fixed values of initial GLY 0.6 M and 0.5 M hydrazine at pH 9.2, it was difficult to control the relative concentrations of GLY and hydrazine to maintain the identical pH values and reaction conditions.

GLY exhibited an increasing Raman intensity correlated to [Cu^2+^] in water (50 µM) on AuNPs. Response behaviors were examined for GLY in the potential interference of various metal ions on AuNPs. The Raman spectral features of GLY-M^n+^ on AuNPs were unchanged in the presence of Fe^3+^, Fe^2+^, Hg^2+^, Mg^2+^, Mn^2+^, Ni^2+^, Zn^2+^, Cr^3+^, Co^2+^, Cd^2+^, Pb^2+^, Ca^2+^, NH_4_^+^, Na^+^, and K^+^ (all at concentrations of 50 µM). Of all the tested metal ions, only Cu^2+^ ions increased the Raman intensity in the presence of GLY on AuNPs.

Inductively coupled plasma optical emissions spectrometry (ICP-OES) was used to estimate the atomic compositions of the prepared samples with an ICAP-7400 analyzer from Thermo Scientific (Waltham, MA, USA) except the Hg measurements by a CETAC M-7500 mercury analyzer from Parma Company (Omaha, NE, USA). Triply-distilled water was used to test the ionic detection. In order to check the applicability of our analytical methodology, the river water samples were obtained from Han river (Seoul, Korea), respectively. Raman spectra for ionic detection in environmental samples were obtained as in the method of our recent report [24,25,26].

## 3. Results and Discussion

### 3.1. Physical Characterization

**Figure 2:**
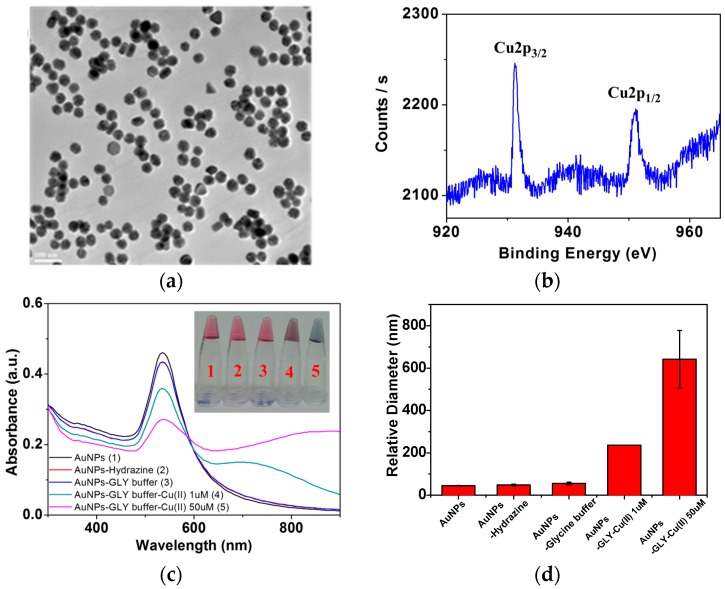
(**a**) TEM image and (**b**) XPS spectrum of GLY-Cu^2+^ on AuNPs; (**c**) UV-Vis absorption spectra. The inset shows a photo of the AuNP solutions in the (1) absence and (2) presence of hydrazine, (3) glycine buffer and glycine buffer-Cu^2+^ with [Cu^2+^] = 1 and 50 µM in (4) and (5), respectively; (**d**) Stick diagram of relative hydrodynamic diameters of GLY-Cu^2+^ on AuNPs in the [Cu^2+^] concentration range of 1 and 50 µM.

To understand the surface reactions, AuNPs were characterized by HR-TEM and XPS. The sizes of the AuNPs are ~40 nm, as shown in the TEM image in Figure 2a. We could also find a trace amount of the Cu species on AuNPs by EDAX. The relative abundance of the adsorbed Cu species on metal NPs could be found to be as low as 0.2%–0.6%. As shown in Figure 2, XPS data [27] also confirmed that the percentage of Cu is as low as 0.2%. Because of a small amount of the Cu species below 1%, we could not map the Cu distribution on a single nanoparticle.

By adding GLY, the surface charge became slightly more negative. Zeta potential measurements indicated that the surface charges appeared to become positive and almost neutral to destabilize the colloidal conditions and induce aggregation in the presence of Cu^2+^ ions, as is consistent with the UV-Vis data. This result suggests that the high concentration of may oxidize the GLY complex on the metal nanoparticle surfaces. On the basis of the surface characterization on AuNPs, we attempted to monitor Cu^2+^ ions by Raman spectroscopy.

Figure 2c shows the color change of AuNPs upon the increase of the Cu^2+^ ion concentrations. As the concentration of Cu^2+^ ions increased in the complexes, the UV-Vis absorption band at 600–900 nm became stronger, indicating the aggregation of AuNPs. It has to be mentioned that AuNPs intrinsically have negative charges. As shown in Figure 2c, the SPR bands of AuNPs are newly presented instead of those of AgNPs. The decrease in the SPR bands at 520 nm may be caused not by the destruction of the nanoparticles but by the increase in particle sizes owing to aggregation. Instead, we observed extensive shifts of the SPR band between 600 and 900 nm to support the evidence of interparticle aggregation.

To address the aggregation issue, we newly performed the dynamic light scattering measurements as shown in Figure 2d. Since the static TEM measurements may not provide good information on the aggregation during the sample preparation process, as solution evaporation may induce particle aggregation owing to dryness on the sample grid, we performed light scattering measurements to estimate the hydrodynamic diameters of the aggregated AuNPs in aqueous solutions to better address the issue of aggregation in relation to SERS intensities. At [Cu^2+^] = 1 and 50 µM, the diameters became 240 and 640 nm, respectively, to suggest an extensive aggregation mediated by the Cu ion species, which should result in strong SERS effects.

### 3.2. Raman Spectra of Hydrazine and GLY on AuNPs

**Figure 3:**
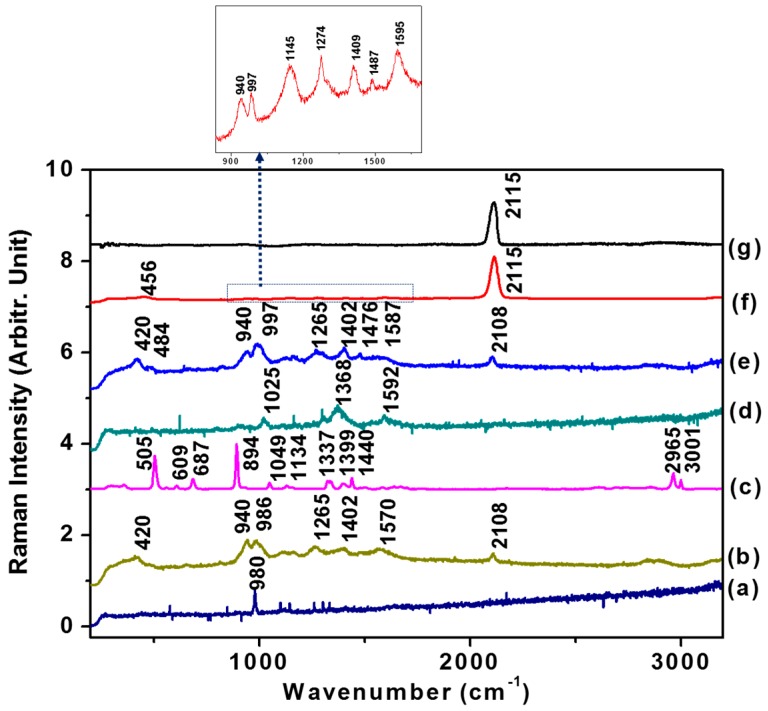
(**a**) NR of and (**b**) surface-enhanced Raman scattering (SERS) of hydrazine (wt. 35% in H_2_O) on AuNPs; (**c**) NR of GLY; (**d**) SERS of GLY on AuNPs; (**e**) SERS of GLY (hydrazine buffer) with [Cu^2+^] = 0; (**f**) SERS of Gly (hydrazine buffer) with [Cu^2+^] = 50 µM. The inset shows a magnified view of the weak vibrational bands of hydrazine; (**g**) SERS spectrum of 0.1 M KCN on AuNPs.

Figure 3 shows the normal Raman (NR) and SERS spectra of GLY on AuNPs. The Raman spectrum of GLY is consistent with that reported previously [28,29,30]. It was found that most strong bands could be ascribed to the hydrazine peaks, as previously reported [31,32,33]. The SERS spectrum of GLY appeared to be relatively weak with its amino group interacting with Au surfaces. In the SERS spectrum of hydrazine on AuNPs, we could not observe a strong hydrazone linkage-related N-N stretching mode at 919 cm^−^^1^ or N-H bending modes at 1174 and 1222 cm^−^^1^ [34].

Since hydrazine shows weak basic properties in aqueous solution to form stable salts with a number of mineral acids such as hydrazine sulfate N_2_H_5_·HSO_4_ and hydrazine hydrochloride N_2_H_4_·HCl, it may interact with the GLY amino acid efficiently. As suggested from the SERS spectrum of GLY in the presence of hydrazine as shown in Figure 3e, GLY can be decomposed to yield a CN peak even in the absence of Cu^2+^. A magnified view of Figure 3f in the presence of Cu^2+^ also exhibited the hydrazine peaks. As a control experiment without hydrazine, we could not observe any CN peaks. This result indicates a role of hydrazine to bind the amino acid as a base after adsorption on the metal nanoparticle surfaces. The other amino acids including methione, lysine, and leucine would not provide strong CN peaks as in the case of GLY. The simple structure of GLY along with the strong interaction with the Cu(II) ion may facilitate its dissociation to yield CN peaks on metal surfaces, whereas the reaction appears to be unfavorable to the other amino acid. Considering the high initial concentrations of both GLY (0.6 M) and hydrazine (0.5 M), we cannot exclude the interactions between hydrazine and Cu^2+^. As suggested from the SERS features of hydrazine in Figure 3f, it is likely that hydrazine adsorbed fairly strongly on Au to interact mainly with GLY. The increase of the Cu^2+^ ion was thought to lead a stabilization of the hydrazine-modified AuNPs.

It is noteworthy that the CN band at ~2108 cm^−1^ was newly appeared in the SERS spectra. In the presence of 50 µM of Cu^2+^ ions, the CN band became more intensified. The band position at ~2108 cm^−1^ corresponds to the CN species adsorbed on Au surfaces, considering that this is almost identical to the CN band at 2115 cm^−1^ when 0.1 M KCN is treated on AuNPs, as demonstrated in Figure 3g.

Our result is consistent with the stoichiometric reaction [20] equations that the CN group may be formed on the surfaces under alkaline conditions. Furthermore, the decomposition of GLY at the positive surface charge is in agreement with this report [20]. Referring from the previous reports of the formation constants [35,36] of GLY-Cu and metal-CN, it was expected that CN would strongly coordinate to the Cu ions. However, considering that only less than 1% of Cu(II) ions exist on nanoparticle surfaces in XPS measurements as suggested in Figure 2b, most CN bands may be ascribed to the AuCN mode.

**Figure 4:**
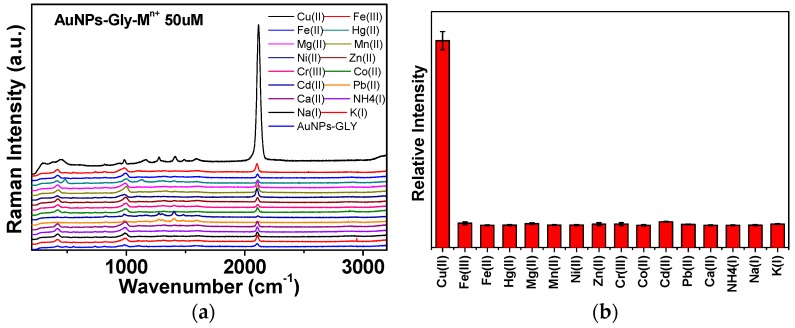
(**a**) SERS spectra of GLY in the presence of 50 µM of various ions. The SERS spectrum of GLY on AuNPs is presented in the middle. Only the Cu^2+^ ion could induce a strong CN peak as listed at the top of the spectra; (**b**) Stick diagram of the relative intensities of the CN bands for the tested ions. Three independent measurements were performed to yield the error bars in order to check the reproducibility.

We obtained the SERS spectra of GLY on alkaline AuNPs in the presence of various ions—Fe^3+^, Fe^2+^, Hg^2+^, Mg^2+^, Mn^2+^, Ni^2+^, Zn^2+^, Cr^3+^, Co^2+^, Cd^2+^, Pb^2+^, Ca^2+^, NH_4_^+^, Na^+^, and K^+^—at the same concentrations of 50 µM, in comparison with Cu^2+^ as shown in Figure 4a. It was found that only Cu^2+^ ions strongly enhanced CN formation as illustrated in the stick diagram of Figure 4b. It is reported that Au and Cu can form a bimetallic structure [37]. Presumably, due to similar crystal structures and electronic properties, the Cu^2+^ ion may easily adsorb on AuNP surfaces instead of the other ions.

### 3.3. CN Formation Reactions of GLY-Cu^2+^ on AuNPs and Selective Test

**Figure 5:**
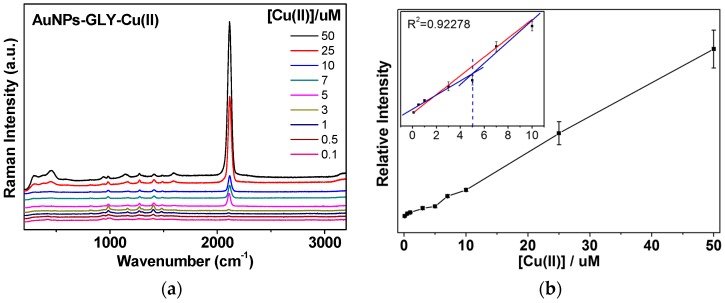
(**a**) Concentration-dependent SERS spectra of GLY depending on the [Cu^2+^] concentration. Baseline corrections were made to compare the spectral bands; (**b**) Intensity plot of the CN intensities versus the [Cu^2+^] concentration of 0–50 µM. The inset shows fitting the linear region between 0 and 10 µM.

Figure 5 shows SERS spectra of GLY depending on the Cu^2+^ concentration. It seems certain that the band at ~2108 cm^−1^ can be ascribed to the CN species, referring from the unique band position. The CN stretching region depending on the Cu^2+^ concentrations was magnified between 2000 and 2200 cm^−1^ in Figure 5a. It revealed an increase in the band intensities depending on the Cu^2+^ ion concentration. It was found that the CN stretching band showed a rather asymmetric shape that could be decomposed into two bands at ~2070 and ~2108 cm^−1^. It is not absolutely certain whether the weaker band may be ascribed to the CN mode on Cu and Au surfaces, depending on their close vibrational band positions [38]. Considering that only KCN also exhibited an asymmetric shape, the weaker shoulder peaks may suggest a different binding mode on Au surfaces. In the previous study [17], the peak positions of CN band appeared to blueshift as the applied potential become more positive. The CN peak became blueshifted from ~2108 cm^−1^ to ~2115 cm^−1^, as the concentrations of the Cu^2+^ ion increased, presumably due to more cationic environments. 

Figure 5b shows an intensity plot of the CN stretching bands depending on the [Cu^2+^] concentration range of 0–50 µM in river water. The detection limit on the basis of the CN band is as low as 2 µM of Cu^2+^ ions. The proposed detection limit was estimated from the linear fitting curve, where the spectral change could begin to occur based on the linear equation of *Y* (CN intensities) = *B* (intercept) + *A* (slope) × *X* (Cu^2+^ concentrations).

### 3.4. CN Bands in River Water Samples Contacting Highly Concentrated Na, Ca, Mg, and K Ions

**Figure 6:**
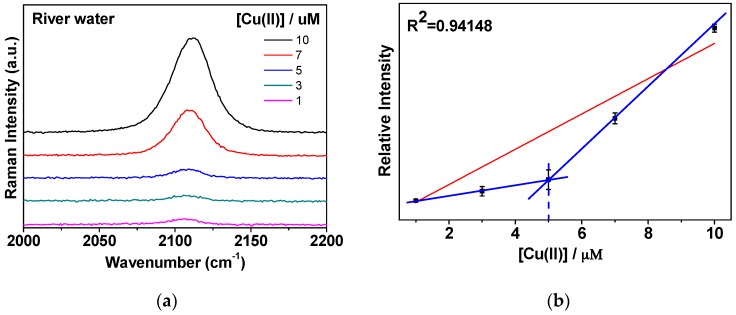
(**a**) A magnified view of the CN stretching region between 2000 and 2200 cm^−1^; (**b**) Intensity plot fitting the linear region on the [Cu^2+^] concentration range of 1–10 µM for river water. Three independent measurements were performed to yield the error bars.

As proof of application, the concentrations of Ca, K, Na, and Mg, were found to be ~31.04 ppm, ~6.37 ppm, ~25.19 ppm, and ~5.77 ppm for the river water sample [24,25,26]. As demonstrated in Figure 6, our analytical methodology of Cu^2+^ determination could be applied in real water samples only with higher limit of detection. It has to be mentioned that ~2 µM is still lower that the EPA recommendation. We could clearly observe the marker band of ~2108 cm^−1^ at micromolar concentrations of Cu^2+^. The detection limit may be expected to improve toward around 1 µM by optimizing the experimental conditions, although there is a turning point to yield a higher slope above ~5 µM.

### 3.5. Cu^2+^ Ion Detection in Cancer Cells

**Figure 7:**
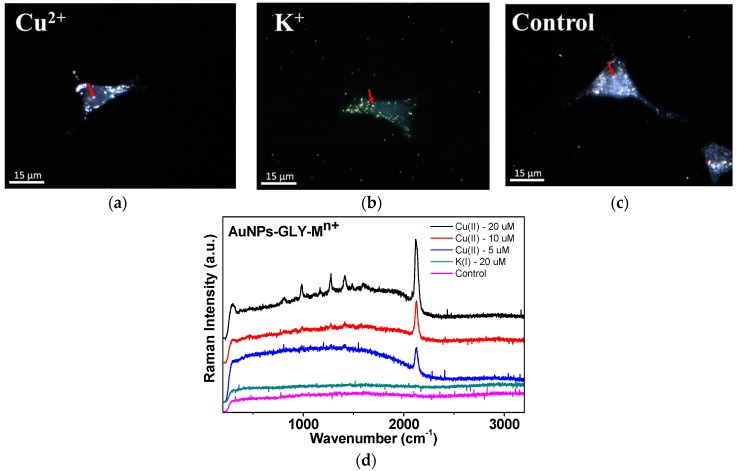
DFM cell images of HeLa cells treated with AuNPs-GLY-Cu^2+^ (20 µM) (**a**); AuNPs-GLY-K^+^ (20 µM) (**b**); and AuNPs-GLY (**c**) for 24 h and subsequently the SERS spectra were observed. Only Cu^2+^ treated cells achieved the CN peak (**d**). The red arrows indicated the positions where the SERS spectra were taken.

We attempted to detect the intracellular concentrations of Cu^2+^ ion in cellular media as depicted in Figure 7. We could observe the increase of the CN peaks in the Cu^2+^ ion concentration ranges between 5 and 20 µM, whereas we could not find any CN peaks for the control tests at 20 µM of K^+^ ion. Since the free Cu^2+^ ion inside cells may be much lower than that of the total copper ions, it is admitted that our methods may be limited in the case of the usages for the intracellular cellular free Cu^2+^ ion measurements under our experimental conditions. 

Our current Raman spectroscopy-based ion detection approach may provide several advantages including better spectral resolution and multiplexing, which can be applied to the areas of cancer cell research, pharmaceutical identification, and characterization of dynamic interactions between nanoparticles and cells to overcome the limitation of the colorimetric method as depicted in Figure 2c. 

Considering the unique spectral position of the CN band, the potential of our detection method is thought to be promising in comparison with the conventional SERS method, which may suffer from the spectral overlap in the congested wavenumber region. We shall keep improving the sensitivity by introducing novel nanostructures and optimizing the experimental parameters. Our method may provide a unique tool for detecting Cu^2+^ ions in an aqueous solution by means of Raman spectroscopy in a selective and sensitive manner.

## 4. Conclusions

We report a new detection method for Cu^2+^ ions by referring to the CN band increase by means of SERS of the dissociation from GLY on hydrazine-adsorbed AuNPs. UV-Vis absorption spectra are also introduced to check the [Cu^2+^]-induced surface change to indicate the Au particle aggregation to result in the color change. The surface charge variation is characterized by means of zeta potential measurements. The cyanide band at ~2108 cm^−1^ became prominent upon the increase of Cu^2+^ ions. The cyanide peak intensities may be correlated with the Cu^2+^ ion concentration. The other 15 ions of Fe^3+^, Fe^2+^, Hg^2+^, Mg^2+^, Mn^2+^, Ni^2+^, Zn^2+^, Cr^3+^, Co^2+^, Cd^2+^, Pb^2+^, Ca^2+^, NH_4_^+^, Na^+^, and K^+^ have not produced such spectral changes as Cu^2+^. The detection limit based on the CN band is estimated to be as low as 2 µM of Cu^2+^ ions in river water. Our method was found to correlate with the Cu^2+^ ions under intracellular conditions in cancer cells.
